# The intraocular lens position in high myopia eyes

**DOI:** 10.1007/s10384-026-01327-2

**Published:** 2026-02-27

**Authors:** Ziyi Wang, Siqing He, Ning Ding, Chen Xin, Zhe Dong

**Affiliations:** 1 Hebei Provincial Eye Hospital, Hebei, 054001 Xingtai China; 2 Beijing Tongren Hospital, Capital Medical University, Beijing, 100730 China; 3 Ophthalmology Department, Beijing Tongren Hospital, Capital Medical University, Beijing, 100730 China

**Keywords:** Intraocular lens, Decentration, Tilt, High myopia, Anterior segment optical coherence tomography

## Abstract

**Purpose:**

To explore the positional relationship between intraocular lens (IOL) and crystalline lens (CLL) by anterior segment optical coherence tomography (AS-OCT) in high myopia complicated with cataract eyes.

**Study design:**

Retrospective and consecutive study.

**Methods:**

Patients who had undergone phacoemulsification with IOL implantation were reviewed. Exclusion criteria were any ocular history that could have affected the zonules, poor pupil dilation and intra- or post- operative complications. The magnitude and direction of tilt and decentration of CLL and IOL, CLL thickness (LT), anterior chamber depth (ACD) and axial length (AL) were measured.

**Results:**

Fifty eyes without high myopia (EM group) and 73 high myopia eyes (HM group) were recruited. The magnitude of tilt and decentration of CLL was significantly different between the HM and EM group (p_tilt HM vs. EM_ <0.01, p_decentration HM vs. EM_ <0.01). In terms of the decentration, the magnitude of IOL was significantly higher than of CLL in both the HM and EM groups (p _HM_ < 0.01; p _EM_=0.043). Multivariate analysis showed that only the alignment of CLL was related with the position of IOL in both group (R^2^_tilt EM_=0.237, p _tilt EM_ < 0.01, R^2^_decentration EM_=0.097, p _tilt EM_=0.042; R^2^_tilt HM_=0.476, p _tilt HM_ < 0.01, R^2^_decentration HM_=0.359, p _tilt HM_ < 0.01).

**Conclusions:**

Alignment of CLL in high myopia eyes determines the position of the IOL. Moreover, the IOL in high myopia was more decentrated, while this discrepancy did not reach a level that would compromise visual quality, it needs to be taken into consideration.

## Introduction

With the development of medical care and improvement of living standards, cataract surgery is performed not only for the restoration of visual acuity but also for achieving the best visual quality. The misalignment of intraocular lenses (IOL) is an important factor interfering with visual quality after cataract surgery.

It is reported that tilt and decentration are the best parameters that present the alignment of either the crystalline lens (CLL) or the IOL. The alignment of the IOL could bring about a higher order aberration, especially for aspheric, toric and multifocal IOLs [1–11]. The number of patients with high myopia increases year by year, especially in China [12, 13]. It is assumed that long axial length (AL) might increase the instability of the IOL position, resulting in more rotation as well as poorer zonule stability [14, 15]. Thus, some studies propose that it is not suitable for patients with high myopia (HM) to have multifocal IOLs implanted [13, 15, 19].

Many studies demonstrate that the initial position of CLL is strongly correlated with IOLs misalignment [16–18]. However, few articles had characterized the lens position of patients with high myopia and cataract. China has the largest number of people with HM and it is increasing. This study seeks to investigate the factors that influence IOL location in patients when combined with high myopia and cataract.

## Methods

### Patients

Consecutive cases of age-related cataract, scheduled to receive phacoemulsification with IOL implantation in the ophthalmology department of Beijing Tongren Hospital between June 2023 and January 2024 were recruited retrospectively. The exclusion criteria were: a history of ocular surgery, trauma or disease that potentially affected the zonules, such as uveitis, glaucoma or pseudoexfoliation syndrome; patients with poor pupil dilation disturbing the capsulorhexis; patients with intra- or post- operative complications, such as posterior capsule rupture and broken zonular fibers. The study was approved by the Beijing Tongren Eye Center Ethics Committee (U20A20196), adhered to the tenets of the Declaration of Helsinki. Written informed consent was signed by the individual recruited subjects.

### Comprehensive ocular examination

Patient data, including age, gender, and medical history, were obtained from the patients’ medical records. A complete ophthalmologic examination was performed preoperatively. The examination included best corrected visual acuity (BCVA), slitlamp evaluation, non-contact tonometer (Canon TX-20), fundoscopy, corneal topography, IOLMaster 700 (Carl Zeiss Meditec AG) and anterior segment optical coherence tomography (AS-OCT, SS-1000, Tomey Casia 2) evaluation. Preoperative ocular biometric parameters were measured by IOLMaster 700 including AL.

### AS-OCT examinations

**Fig. 1:**
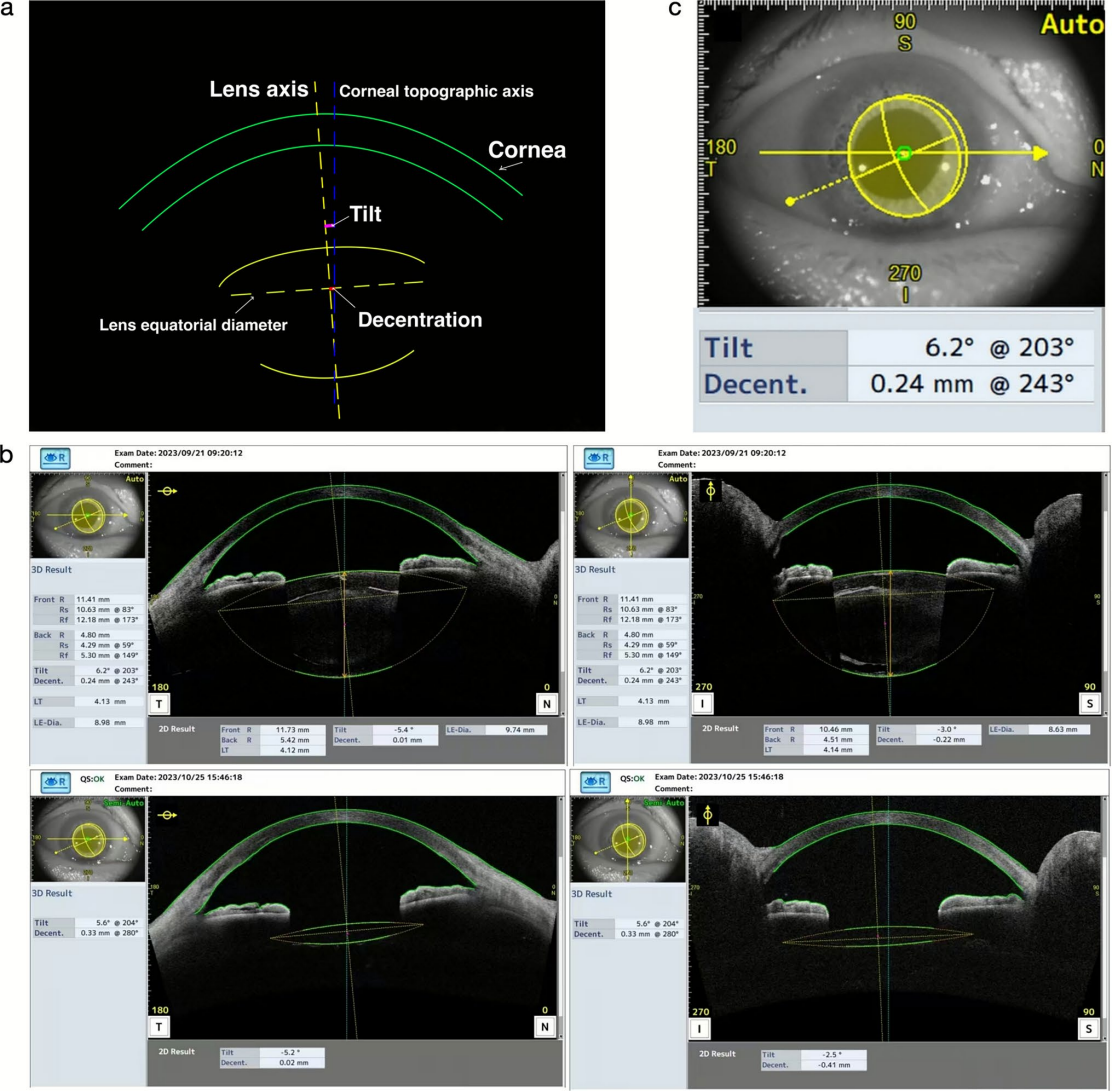
CASIA 2 automatically measured the tilt and decentration of lens. **a** The measurement principle of anterior segment optical coherence tomography (AS-OCT) about lens is shown in the simple 2D picture. The green curves simulate the front surface and back surface of cornea. The yellow curves simulate the front surface and back surface of the lens. The yellow dotted lines simulate the lens equatioual diameter and lens axis. The blue dotted line simulates the corneal topographic axis. The red line shows the decentration of lens. The pink curve shows the tilt of lens. **b** Above: the horizontal and vertical results preoperatively; below: the horizontal and vertical results postoperatively. **c** The 3D picture shows that in 3D result, the tilt of lens is recorded as ‘6.2°’ and the decentration of lens is recorded as ‘0.24 mm’

Non-mydriatic AS-OCT imaging (CASIA 2) was conducted before and one-month after cataract phacoemulsification and IOL implantation. One single, masked and experienced examiner obtained all images under identical standard lighting conditions. The CASIA 2 could automatically measure the tilt and decentration of the crystalline lens or the IOL relative to the corneal topographic axis (Fig. 1 A). The directions of tilt and decentration for CLL or IOL are based on a coordinate system in which the 0° is located on the observer’s right and 90° in the superior direction (Fig. 1 B). All tilt data are presented as a (°)@ b (°); at direction b, the tilt of the CLL or IOL has the largest magnitude equal to ‘a’. Decentration data are presented as c (mm)@ d(°), which means that at direction ‘d’, the decentration of the crystalline lens or IOL has the largest magnitude equal to ‘c’ (Fig. 1 C). Individual tilt (°) or decentration (mm) and its azimuth (°) values were transformed into Cartesian coordinates (x and y) using the method described by Holladay et al. [19, 20], which validated the accuracy and uniqueness of the lens position (Fig. 1). In this study, our primary focus was on the documentation and quantitative analysis of tilt and decentration magnitudes.

### Cataract surgery

All cases underwent cataract phacoemulsification with IOL implantation surgery under local anesthesia. A self-sealing 2.4 mm temporal corneal incision was done. After continuous circular capsulorhexis and cataract phacoemulsification, one-piece hydrophobic IOL (A1UL22; Eyebright Medical Technology Co., Ltd.) was implanted and the alignment of IOL was ascertained. All surgical procedures were performed by DZ.

### Statistical analysis

All statistical analyses were performed using SPSS for Mac, version 27.0 (IBM Corp.,). p value < 0.05 was considered significant. Baseline data are presented as mean ± standard deviation (SD). T test was used for the comparison of data. A higher R-squared value larger than 0.1 indicates higher fitting and vice versa. Regression coefficients (RCs) values ranged from -1 to 1. A higher RC absolute value close to 1 indicated higher reliability and vice versa.

## Results

123 eyes of 88 patients (50 eyes in the group without high myopia (EM) and 73 high in the HM group) were included in this study. The demographic and ocular characteristics are presented in Table 1. This study included 36 men and 52 women; the mean age of the EM group was 64.83±10.23 years old and the mean age of HM group was 56.07±8.48 years old. The AL was 23.81±1.21mm in EM group and 29.52±2.28mm in HM group. The mean tilt was 4.84° ± 1.73° preoperatively and 4.80° ± 1.67° postoperatively in EM group and 3.51° ± 2.20° preoperatively and 3.41° ± 1.62° postoperatively in HM group. The mean decentration was 0.15 ± 0.08mm preoperatively and 0.18 ± 0.13mm postoperatively in EM group and 0.22 ± 0.13mm preoperatively and 0.30 ± 0.17mm postoperatively in HM group.

**Table 1:** Demographic and ocular characteristics

	**EM**	**HM**	*p* value
Patients, n	41	47	-
Male, n	12	24	-
Female, n	29	23	-
Age (yrs.), mean (SD)	64.83±10.23	56.07±8.48	<0.001*
Eyes, n	50	73	-
Right eyes, n	27	38	-
Left eyes, n	23	35	-
AL (mm), mean (SD)	23.81±1.21	29.52±2.28	<0.001*
ACD (mm), mean (SD)	2.74±0.50	3.07±0.36	<0.001*
LT (mm), mean (SD)	4.46±0.49	4.40±0.43	0.486
LE-Dia (mm), mean (SD)	9.71±0.85	10.08±0.54	0.004*

AL: Axial length; ACD: Anterior chamber depth; LT: Lens thickness; LE-Dia: Lens equatorial diameter; EM: Emmetropia group; HM: High myopia group

^*^Statistically significant (*p* < 0.01)

The results of Wilcoxon paired test for preoperative CLLs and IOLs tilt and decentration are shown in Tables 2 and 3. There was no significant difference between the CLLs and IOLs in terms of the magnitude of the tilt. The magnitude of decentration increased dramatically after surgery with statistical significance in both the EM (p = 0.043) and HM group (p < 0.01).

**Table 2:** The tilt and decentration of CLLs and IOLs in emmetropia (EM) Group

	**T (°)** **(mean±SD)**	**D (mm)** **(mean±SD)**
CLLs	4.84±1.73	0.15±0.08
IOLs	4.80±1.67	0.18±0.13
P value	0.876	0.043*

T (°): tilt; D (mm): decentration; EM: Emmetropia; HM: High myopia; CLL: crystalline lens; IOL: intraocular lens

*Statistically significant (*p* < 0.05)

**Table 3:** The tilt and decentration of CLLs and IOLs high myopia (HM) Group

	**T (°)** **(mean±SD)**	**D (mm)** **(mean±SD)**
CLLs	3.51±2.20	0.22±0.13
IOLs	3.41±1.62	0.30±0.17
P value	0.670	<0.001*

T (°): tilt; D (mm): decentration; EM: Emmetropia; HM: High myopia; CLL: crystalline lens; IOL: intraocular lens

*Statistically significant (*p* < 0.01)

Univariate and Multivariate analysis of the EM and HM group showed that among the five independent variables related to IOL tilt, the tilt magnitude of CLLs demonstrated a significant linear correlation in both the EM and HM groups (R^2^_tilt EM_=0.237, p < 0.001; R^2^_tilt HM_=0.476, p < 0.001). Additionally, in the HM group, the LT also exhibited a significant linear correlation with IOL tilt (p = 0.002). Meanwhile, among the five independent variables related to the decentration of IOLs, the decentration of CLLs demonstrated a significant linear correlation in both the EM and the HM group (R^2^_decentration EM_=0.097, p = 0.042; R^2^_decentration HM_=0.359; p < 0.001). The aforementioned data demonstrates that tilt and decentration of CLLs is the highest determinant coefficient of IOLs position (Table 4).

**Table 4:** Multivariate analysis of the association between IOLs tilt and decentration and structural parameters in EM group and HM group

**Variables**	**EM group**			**HM group**		
	P value	R^2^	Regression Coefficient	P value	R^2^	Regression Coefficient
**Tilt**						
AL	0.340	0.237	-0.209	0.119	0.476	0.122
ACD	0.394		0.554	0.714		-0.198
LT	0.918		-0.059	0.002**		-1.680
LE-Dia	0.517		0.218	0.999		-0.001
Tpr	<0.001**		0.472	<0.001**		0.454
**Decentration**						
AL	0.768	0.097	0.005	0.765	0.359	0.003
ACD	0.875		0.009	0.568		-0.035
LT	0.398		-0.042	0.202		-0.075
LE-Dia	0.378		0.025	0.209		0.051
Dpr	0.042*		0.467	<0.001**		0.698

Tilt: the tilt of IOLs postoperatively; Decentration: the decentration of IOLs postoperatively; Tpr: the tilt of crystalline lenses preoperatively; Dpr: the decentration of CLLs preoperatively; LT: Lens thickness; LE-Dia: Lens equatorial diameter; AL: Axial length; ACD: Anterior chamber depth

*Statistically significant (*p* < 0.05), **Statistically significant (*p* < 0.01)

There were statistically significant differences between the two groups in terms of amplitude of CLLs and IOLs tilt and decentration as well as the preoperative and postoperative ACD. However, there were no statistically significant differences between the two groups in LT, LE-Dia, variation of tilt degree, variation of decentration degree and variation of ACD (Table 5).

**Table 5:** Comparison of lens position parameters and variations from different AL

**Characteristics**	**Groups**		*p* value
	**EM**	**HM**	
T_pr_ (°)	4.84±1.73	3.51±2.20	<0.001**
D_pr_ (mm)	0.15±0.08	0.21±0.14	0.003**
LT (mm)	4.46±0.49	4.40±0.43	0.486
LE-Dia (mm)	9.71±0.85	10.08±0.54	0.004**
ACD_pr_ (mm)	2.74±0.50	3.07±0.36	<0.001**
T_po_ (°)	4.80±1.67	3.41±1.62	<0.001**
D_po_ (mm)	0.18±0.13	0.30±0.17	<0.001**
ACD_po_ (mm)	3.93±0.42	4.35±0.34	<0.001**
△T (°)	1.31±1.15	1.11±1.48	0.389
△D (mm)	0.84±0.11	0.12±0.11	0.017*
△ACD (mm)	1.20±0.40	1.27±0.28	0.232

T_pr_ (°): preoperative tilt; D_pr_ (mm): preoperative decentration; T_po_ (°): postoperative tilt; D_po_ (mm): postoperative decentration. All the data were presented as mean±SD

LT: crystalline lens thickness (mm); LE-Dia: Lens equatorial diameter; ACD: anterior chamber depth(mm); AL: axial length (mm); △T= postoperative tilt-preoperative tilt (the variation of tilt); △D=postoperative decentration-preoperative decentration(the variation of decentration)

*Statistically significant (*p* < 0.05), **Statistically significant (*p* < 0.01)

## Discussion

HM is an increasing ocular disease worldwide. In China, it has become and is a cause for concern. With the increase in HM patients that also have cataracts, the challenge is how to improve the postoperative visual quality of these patients. It is, therefore, important for ophthalmologists to investigate how to choose the ideal IOLs for HM patients combined with cataract.

Alignment of the IOL is the most important factor affecting visual quality after cataract surgery. Tilt and decentration are the indicators most frequently used to evaluate IOL alignment. Previous literature speculates that long axial length might decrease the position stability of the intraocular lens, resulting in more rotation as well as poorer zonule stability [14, 15], which are the causes of postoperative IOL displacement. However, our study shows that there was no significant correlation between AL and the degree of postoperative IOL displacement, and the position of postoperative IOL depends more on the position of the preoperative CLL. We speculate that different operating habits as well as phacoemulsification energy using habits of different surgeons may affect the IOL position of in HM eyes following cataract surgery.

In this study, the preoperative and postoperative lens position characteristics of patients with HM combined with cataract were recorded by AS-OCT and compared with those of the normal AL population. The position misalignment of IOLs in cataract patients with high myopia mainly depends on the position misalignment of the CLLs; tilt was not affected by the length of the AL, and even showed a decrease with the growth of AL. It has been consistently believed that the AL has a strong positive relationship with IOL’s tilt compared to CLL’s, which has not been observed in our study. This may be due to the fact that the longer AL concentrates on the elongation of the posterior pole of the eye and has little or no effect on the stretching of the anterior face of the eye [18]. Decentration of the IOLs is greater than preoperatively, which may be due to the fact that the transverse diameter of the IOL tends to be smaller than of the CLLs, so that the IOL becomes more mobile in the capsular bag. Zhu et al. also point out that longer ALs have an effect on capsule size, which means the incompatibility between IOLs and capsule size should not be underestimated in HM eyes [19]. Tokuhisa et al. suppose that the IOLs often have a larger space for movement compared to CLLs [20–22].

Our research has many limitations. First, the limited number of participants. It needs to be expanded to make the data more independent in subsequent research. In this study, we focused on the comparison between the EM and HM groups. Our observations show that the mean age of the HM group in this study was significantly younger than of the EM group. Upon reviewing the ophthalmological examination results, we hypothesize that this discrepancy arises from the fact that the HM participants predominantly underwent cataract surgery due to severe nuclear lens opacity causing visual impairment, whereas the EM participants primarily experienced vision problems associated with age-related cortical lens opacity. These objective findings should be systematically documented in subsequent research using the Emery-Little classification to elucidate the underlying mechanisms contributing to the observed age differences between these distinct cohorts. Second, our current research remains primarily focused on the processing and analysis of examination data, with a notable absence of objective evidence regarding patients' visual quality assessment. Numerous factors could influence the postoperative visual quality of patients, necessitating further refinement and supplementation in subsequent research. Moreover, due to the methodological constraints of AS-OCT's bilateral eye measurements conducted separately, the obtained results exhibit slight discrepancies from the patient's binocular visual results. Nevertheless, it remains one of the most accurate diagnostic approaches currently available for assessing patients' visual conditions.

Notwithstanding the aforementioned limitations, the position-stable result of IOLs in the HM group is rarely mentioned in the existing literature, this structural characteristic as well as the mechanism that makes the alignment of IOLs stable need to be further studied and explained. Our research indicates that while the magnitude of IOLs decentration in the HM group significantly increased following cataract surgery, the magnitude remains insufficient to induce visual quality impairment. Consequently, a longer AL does not contribute to an additional risk of visual quality issues associated with IOLs decentration post-cataract surgery. Since HM has greater variability in decentration compared to EM, it is recommended to examine the detailed parameters of CLLs using AS-OCT before inserting multifocal intraocular lenses. The decentration and tilt of IOLs are the most frequently observed indicators after the wide application of AS-OCT. The magnitude of decentration or tilt is considered an important factor affecting visual quality. It cannot be denied that the tilt of IOL is naturally accompanied by decentration, to a greater or lesser extent. However, as two sets of data that can be independently quantified by the AS-OCT, they have been widely used in existing studies to discuss and research the position of IOLs. Perhaps with the continuous development of inspection technology, there will be more rigorous measurement indicators for research and discussion in the future. One crucial aspect that cannot be overlooked is that the measurement and collection of parameters mentioned in this paper depend on AS-OCT, a new measuring method that will contribute to better understanding of ocular conditions.

In this study, AS-OCT was used to further explore the influencing factors of IOL location in patients with high myopia complicated with cataract. The study reveals that the relationship between the magnitude of tilt and decentration of IOLs and the AL was determined, the alignment of CLL determines IOL position in HM eyes, filling the current research blind spot.
